# Astrocyte-Mediated Neuromodulatory Regulation in Preclinical ALS: A Metadata Analysis

**DOI:** 10.3389/fncel.2018.00491

**Published:** 2018-12-17

**Authors:** Kathleen Jordan, Joseph Murphy, Anjanya Singh, Cassie S. Mitchell

**Affiliations:** ^1^Laboratory for Pathology Dynamics, Department of Biomedical Engineering, Georgia Institute of Technology, Emory University School of Medicine, Atlanta, GA, United States; ^2^School of Medicine, University of Texas Southwestern Medical Center, Dallas, TX, United States

**Keywords:** glutamate, GABA, GLT-1, ChAT, VGF, TNFα, aspartate

## Abstract

Amyotrophic Lateral Sclerosis (ALS) is a neurodegenerative disease characterized by progressive degradation of motoneurons in the central nervous system (CNS). Astrocytes are key regulators for inflammation and neuromodulatory signaling, both of which contribute to ALS. The study goal was to ascertain potential temporal changes in astrocyte-mediated neuromodulatory regulation with transgenic ALS model progression: glutamate, GTL-1, GluR1, GluR2, GABA, ChAT activity, VGF, TNFα, aspartate, and IGF-1. We examine neuromodulatory changes in data aggregates from 42 peer-reviewed studies derived from transgenic ALS mixed cell cultures (neurons + astrocytes). For each corresponding experimental time point, the ratio of transgenic to wild type (WT) was found for each compound. ANOVA and a student's *t*-test were performed to compare disease stages (early, post-onset, and end stage). Glutamate in transgenic SOD1-G93A mixed cell cultures does not change over time (*p* > 0.05). GLT-1 levels were found to be decreased 23% over WT but only at end-stage (*p* < 0.05). Glutamate receptors (GluR1, GluR2) in SOD1-G93A were not substantially different from WT, although SOD1-G93A GluR1 decreased by 21% from post-onset to end-stage (*p* < 0.05). ChAT activity was insignificantly decreased. VGF is decreased throughout ALS (*p* < 0.05). Aspartate is elevated by 25% in SOD1-G93A but only during end-stage (*p* < 0.05). TNFα is increased by a dramatic 362% (*p* < 0.05). Furthermore, principal component analysis identified TNFα as contributing to 55% of the data variance in the first component. Thus, TNFα, which modulates astrocyte regulation via multiple pathways, could be a strategic treatment target. Overall results suggest changes in neuromodulator levels are subtle in SOD1-G93A ALS mixed cell cultures. If excitotoxicity is present as is often presumed, it could be due to ALS cells being more sensitive to small changes in neuromodulation. Hence, seemingly unsubstantial or oscillatory changes in neuromodulators could wreak havoc in ALS cells, resulting in failed microenvironment homeostasis whereby both hyperexcitability and hypoexcitability can coexist. Future work is needed to examine local, spatiotemporal neuromodulatory homeostasis and assess its functional impact in ALS.

## Introduction

Amyotrophic Lateral Sclerosis (ALS) is a neurodegenerative disease characterized by the progressive degradation of motoneurons, which results in muscle paralysis, respiratory failure, and ultimately death. Its multifactorial nature, population heterogeneity, and inherent complexity has made both clinical survival prediction (Pfohl et al., 2018) and intervention optimization (Khamankar et al., 2018) extremely difficult.

Because the multi-factorial nature of ALS is difficult to study in humans, experimental mouse models are critical for etiological and treatment elucidation. The superoxide dismutase 1 glycine 93 to alanine (SOD1-G93A) transgenic mouse is currently the predominant means of investigating the preclinical pathophysiology of ALS (Pfohl et al., 2015) because it of its rich publication history and reproducibility. ALS pathophysiology has multi-scalar disturbances that impact numerous processes, including inflammation, apoptosis, energetics, excitability, genetic transcription, cellular chemistry, oxidative stress, proteomics, and systemic function (Irvin et al., 2015; Kim et al., 2016). While ALS is a motoneuron disease, non-neuronal tissues are also affected, including astrocytes, glia, and muscle. In fact, inflammation, a process that is predominantly driven by non-neural tissue, is the most published etiology examined in SOD1-G93A ALS mice (Kim et al., 2016).

Evidence suggests that non-neuromuscular cells, such as astrocytes, play one of the earliest roles in ALS and are being considered as therapeutic targets (Vargas and Johnson, 2010; Pehar et al., 2017). Astrocytes are the most abundant subtype of glial cells found in the central nervous system (CNS), comprising 20–50% of the brain volume (Rossi and Volterra, 2009). One function of astrocytes is to stabilize the neural microenvironment after an injury through the release of cytokines (Rossi and Volterra, 2009). Ideally, the inflammatory regulators of astrocytes stimulate the healing process and lay down a protective glial scar (Sofroniew and Vinters, 2010). However, inflammation is also negatively implicated across neuropathology, where its consequences can expand the lesion volume (Mitchell and Lee, 2008) or perpetuate local dysregulation. For example, in SOD1-G93A ALS, there is a dynamic imbalance of cytokines that inappropriately amplifies inflammation (Jeyachandran et al., 2015). Glial fibrillary acid protein (GFAP) levels in SOD1-G93A mice are almost double those of wild type (WT) mice prior to onset and continue to significantly increase with disease progression. Furthermore, ITPR2 gene expression is significantly upregulated before and after the induction of inflammation (Staats et al., 2016).

Another key function of astrocytes is to assist in reuptake of neuromodulatory agents. It has been proposed that the hyperexcitability of motoneurons is caused by overstimulation by the main excitatory neurotransmitter, glutamate, which leads to a large influx of calcium (*Ca2*+) and sodium (Na+) into the cell through glutamate transporter 1 (GLT-1) (Van Den Bosch et al., 2006; Do-Ha et al., 2017). Intracellular *Ca2*+ levels are further increased as Na+ is passed out through the Na+-*Ca2*+ exchanger (Van Den Bosch et al., 2006). Motoneurons possess little ability to counteract the effects of *Ca2*+ influx, so overstimulation can easily lead to excitotoxicity and cell death (Do-Ha et al., 2017). Glutamate excitotoxicity and impaired intracellular calcium signaling in astrocytes is theorized to significantly impact disease progression in ALS and other types of neuropathology (Staat and Van Den Bosch, 2009; Kawamata et al., 2014). Yet, such neuromodulatory regulation mediated by astrocytes is much lesser-studied than inflammation.

Astrocyte neuromodulatory dysregulation, resulting in failed neuromodulatory homeostasis, is thought to contribute significantly to neuronal depolarization, hyperexcitability, excitotoxicity, and subsequent neuronal death in ALS (Lin et al., 2013). Increased levels of glutamate and reduced levels of excitatory amino acid transporter 2 (EAAT2) have been found in the CNS of ALS patients, suggesting EAAT2 dysfunction and glutamate excitotoxicity is involved in the disease progression (Rothstein et al., 1990, 1995). It is still unknown as to what causes the glutamate to initially collect within the CNS. One possible explanation is that, prior to functional onset of the disease, the astrocytes fail to reuptake the extracellular glutamate at the proper homeostatic rate, resulting in a slow accumulation. Another explanation is that the motoneurons, or another nearby cell, releases glutamate at a significantly higher rate than normal. As part of the latter explanation, astrocytes do not compensate for the increased glutamate release and, rather, simply continue to reabsorb at a normal rate, resulting in glutamate accumulation over time. Many studies (Li et al., 2015) have sought to increase GLT-1 levels in astrocytes to increase astrocyte reuptake, albeit unsuccessfully.

The inhibitory neurotransmitter gamma-Aminobutyric acid (GABA) also has therapeutic potential. Mildly reduced expression of GABAergic markers and interneurons have been found in some SOD1 mice and ALS patients (Nihei et al., 1993; Hossaini et al., 2011). GABA transmission can decrease levels of glutamate and protect against excessive neuronal damage (Brockington et al., 2013). However, clinical trials of gabapentin, a pharmaceutical anti-epileptic drug that modulates GABA, did not extend life span or slow the rate of muscle decline or respiratory function (Diana et al., 2017). Elucidation of temporal relationships among glutamate levels, transporter proteins, such as GLT-1 and glutamate receptors (GluR), and GABA is needed to determine therapeutic timing and efficacy. For example, if glutamate-related treatment must occur well before ALS symptom onset to have a functional impact, its clinical treatment value is greatly diminished.

The adjunctive regulation of intracellular *Ca2*+ by astrocytes is vital for cell signaling. Astrocytes are able to signal neurons by *Ca2*+ dependent release of glutamate (Appel et al., 2001; Rossi and Volterra, 2009). Intracellular concentrations of *Ca2*+ are characteristically elevated in response to pathological signaling (Rossi and Volterra, 2009; Guerra-Gomes et al., 2018). The glutamate-mediated excitotoxicity of astrocytes relies on intracellular concentrations of *Ca2*+ but has also been found to be accompanied by the cytokine TNFα (Rossi and Volterra, 2009). The blockage of the formation of TNFα and endoplasmic reticulum (ER) *Ca2*+ overload have a significant negative effect on astrocyte glutamate release (Rossi and Volterra, 2009; Kawamata and Manfredi, 2010). Other cytokines, such as VGF nerve growth factor (VGF), insulin-like growth factor 1 (IGF-1), aspartate, and choline acetyltransferase (ChAT) can also be linked to intracellular calcium levels (Palmieri et al., 2001; Fernández et al., 2004; Kandinov et al., 2013).

An examination of dynamic, temporal interactions among key players in astrocyte-mediated neuromodulatory regulation is necessary to better evaluate ALS etiology and therapy. Of course, pathological extracellular increases of glutamate, calcium, and other co-factors does lead, or minimally contribute, to co-existing pathology, like oxidative stress, inflammation, and excitotoxicity. Such pathological overlap makes detangling the multi-factorial ALS etiology all the more difficult (Kim et al., 2016). Due to the large number of variables, a single all-encompassing *in vivo* experiment is not feasible. The goal of this study is to determine temporal trends of intrinsic astrocyte-mediated compounds that contribute to neuromodulatory regulation over the disease progression of preclinical ALS, with the primary focus on regulation of glutamate, GABA, and related compounds. Specifically, this metadata analysis is comprised of temporal neuromodulatory data compiled from 42 peer-reviewed studies that utilized mixed cultures of astrocytes and neurons predominantly derived from *in vivo* SOD1-G93A ALS mice and normal WT mice.

## Methods

A metadata analysis was performed to construct a macroscopic view of astrocyte-mediated neuromodulatory regulation over the course of transgenic ALS mouse model disease progression. The general method involved (1) mining, selecting and recapturing published data from preclinical ALS experiments examining astrocytes; (2) normalizing recaptured data to enable aggregation across studies; (3) analyzing aggregate data using appropriate statistical methods.

### Data Source Identification and Inclusion Criteria

Keywords were used to identify potential data sources in PubMed/Medline. All potential data sources were initially searched using key words “Amyotrophic Lateral Sclerosis” OR “ALS” AND “transgenic mouse.” Searches were limited to articles published in English and with publication dates through June 2018. Primary search articles were downloaded into a Filemaker Pro relational database (Mitchell et al., 2015a; Kim et al., 2016) and had relevant data recaptured using our lab's highly accurate biocuration process (Mitchell et al., 2015a). Secondary searches on relevant sub-topics were performed within the Filemaker Pro database including all synonyms (see Table 1). We specifically utilized the “high-copy” transgenic SOD1-G93A mouse model, which is not only more common but also has less implicit outcome heterogeneity (Pfohl et al., 2015). Recaptured data included measures of glutamate, GTL-1, GluR1, GluR2, GABA, ChAT activity, VGF, TNFα, aspartate, and IGF-1, which were identified using searches of figure axis labels and figure/table captions. As astrocytic compensation can only be studied in the presence of neurons, most data was taken from mixed cultures. Only studies that presented quantified data for both transgenic and age-matched WT control mice were included. A total of 42 articles with quantifiable experimental data had data extracted for analysis. A diagram of the literature review structure is shown in the Supplementary Figure 1.

**Table 1:** Keywords for “*Astrocytes*” and terms associated with each subtopic.

**Category**	**Keywords**
Glutamate	Nitric oxide, NO^*^ conc^*^, synth^*^, glutamate, glutamate^*^ conc^*^, GLT1, GLT1^*^ transporter^*^, GluRA, GluR1, GluR2, GluR3, GluR4, GluR^*^, excitotoxicity, excito^*^
Calcium	Membrane potential, cyto^*^ cal^*^ conc^*^, cal^*^ buffer^*^ capacity, mito^*^ cal^*^ conc^*^, cal^*^ uptake, cal^*^ conc^*^, cal^*^ transient, permeability transition pore, acetylcholine, voltage-gated, calcium channel^*^, calcium pump^*^, GABA, ER([Ca]), endoplasmic reticulum, AMPA receptor^*^, VDAC, CypD, FCCP, IGF-1, VGF, TNF-alpha, TNF^*^, aspartate, ChAT^*^

*The subtopics were broad topics that were used to categorize articles discussing ALS studies. The keywords were used in searches in the database to determine article numbers and potential data points for each subtopic. The searches were performed on figure axis labels and captions*.

* *indicates that pieces of the word/phrase or synonyms were included in the search*.

### Data Normalization and Aggregation

Recaptured quantified data was used to construct ratios of ALS-representative mouse models to WT (such as, SOD1-G93A/WT) for each included metric at each temporal disease stage. The genetic background, onset, survival, tissue origin/measurement procedure of each included study is detailed in Supplementary Table 1. Temporal data points were aggregated into three disease stages; pre-onset (0–96 days), post-onset (97–116 days), and end stage (117+ days). Disease stages were determined by finding the average age of onset (97 days) and survival duration (117+ days) for the included mice in the present study. Non-aggregated raw data can be found in Supplementary Figure 2. Six of the post-onset glutamate data points were empirically extrapolated using standard statistical regression to obtain the necessary sample size determined by a standard power analysis. Table 2 illustrates the breakdown of the 264 data points (e.g., ratios of transgenic to WT for each included factor for each temporal disease stage). Aggregation is an inherent limitation to meta-analysis, and this is further discussed in the context of this study in the Limitations.

**Table 2:** Number of data points used in each sub-category.

**Sub-Category**	**Time bin**	**Sample size**	**References**
Glutamate	Pre-onset Post-onset End-Stage	22 12^*^ 15	Alexander et al., 2000; Guo et al., 2000, 2003; Bendotti et al., 2001; Raiteri et al., 2004; Niessen et al., 2007; Choi et al., 2009; Gu et al., 2010; Milanese et al., 2011; Albano et al., 2013; Valbuena et al., 2016; Tefera and Borges, 2018
GLT-1	Pre-onset Post-onset End-Stage	22 10 16	Alexander et al., 2000; Bendotti et al., 2001; Deitch et al., 2002; Chen et al., 2004; Rothstein et al., 2005; Boston-Howes et al., 2006; Pardo et al., 2006; Yang et al., 2009; Gu et al., 2010; Benkler et al., 2013; Morel et al., 2013
GluR1	Pre-onset End-Stage	24 7	Petri et al., 2005; Spalloni et al., 2006; Martinez et al., 2008; Zhao P. et al., 2008 Caioli et al., 2011
GluR2	Pre-onset End-Stage	17 5	Petri et al., 2005; Spalloni et al., 2006; Tortarolo et al., 2006; Zhao P. et al., 2008
GABA	Pre-onset End-Stage	4 11	Raiteri et al., 2004; Tsai et al., 2010; Caioli et al., 2013; Tefera and Borges, 2018
ChAT Activity	Pre-onset Post-onset End-Stage	24 12 23	Crochemore et al., 2005, 2009; Kalmar et al., 2012
VGF	Pre-onset End-Stage	12 6	Zhao Z. et al., 2008
TNFα	Pre-onset End-Stage	8 3	Fang et al., 2010; Yang and Cheng, 2010; Song et al., 2013; Cai et al., 2015; Jeyachandran et al., 2015; Lee et al., 2015
Aspartate	Pre-onset	7	Alexander et al., 2000; Niessen et al., 2007; Choi et al., 2009; Tefera and Borges, 2018
IGF-1	Pre-onset	4	Kaspar et al., 2005; Wu et al., 2006; Messi et al., 2007; Fergani et al., 2011

* *To reach the desired sample size, 6 of the 12 data points were linearly extrapolated for this time bin*.

Not every included journal article expressed the mouse sample size corresponding to each of their data points. Thus, each data point (e.g., ratio of transgenic to WT for the included factor measured at a specific temporal disease stage) in the present metadata analysis is weighted equally on a per-article basis rather than the corresponding mouse sample size comprising the data point.

To determine the ALS disease stages, the average onset (97 days) and time of death (117 days) was calculated using all included onset and time of death data from the original experimental studies (see Supplementary Data Sheet). Note that these averages are within what is expected for high copy SOD1-G93A ALS mice (Pfohl et al., 2015).

### Statistical Analysis

The distributions of all data sets were tested with Shapiro-Wilks tests to assure sufficient distribution normality for the corresponding statistical tests. To determine statistical significance of differences between disease stages, an ANOVA was performed using the Bonferroni correction. To compare protein levels, a Student's *t*-test with a Bonferroni correction was performed for each disease stage. Error bars on figures correspond to the standard deviation. All statistical tests were performed with built-in functions in MATLAB (version R2016a). Correlation matrices were constructed to visualize the relationships between the analyzed measures. A principal component analysis (PCA) was used to evaluate contributions to data variance and visualized reduced data structure. The correlation matrices and PCA were generated in MATLAB (version R2016a).

## Results

A total of nine astrocyte-mediated neuromodulatory regulators were examined from 42 peer-reviewed articles. As stated in the Introduction, nearly all of these factors still have secondary ties to inflammation. However, the focus of the present study was not on the inflammatory roles of astrocytes but rather neuromodulatory regulation of GABA, glutamate and calcium related co-factors. Table 2 lists the measures along with the included sample size (number of data points). The glutamate excitotoxicity sub-categories included glutamate, GLT-1, GluR1, GluR2, and GABA concentrations. The calcium homeostasis sub-categories included ChAT activity, VGF, TNFα, aspartate, and IGF-1 levels.

### Glutamate and Glutamate Transporter Proteins

**Figure 1:**
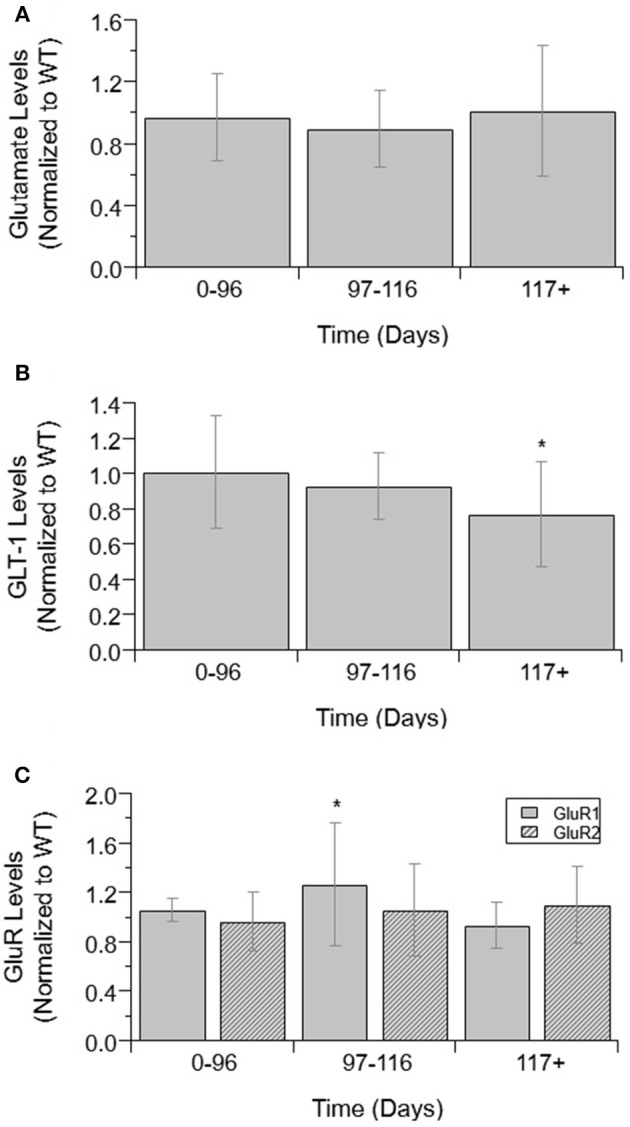
Glutamate, GLT-1, and GluR complex levels in mixed cultures over ALS disease progression**. (A)** Glutamate level average in SOD1-G93A mice normalized to wild-type (ratio presented as SOD1-G93A/WT) in three temporal stages: 0–96 days, 97–116 days, and 117+ days. There was no significant change in glutamate levels between any of the temporal stages. **(B)** GLT-1 average in SOD1-G93A normalized to wild-type (ratio presented as SOD1-G93A/WT) in three temporal stages: 0–96 days, 97–116 days, and 117+ days. GLT-1 is significantly decreased from WT (^*^p = 0.0075) at 117 + days. **(C)** GluR1 (solid bars) and GluR2 (stripped bars) average in SOD1-G93A normalized to wild-type (ratio presented as SOD1-G93A/WT) in three temporal stages: 0–96 days, 97–116 days, and 117+ days. There was no significant change in GluR2 levels between temporal stages. GluR1 at 117+ days was significantly decreased from 0–96 (^*^p = 0.0346). Error bars represent SD.

Glutamate and glutamate transporter levels from mixed cell cultures from SOD1-G93A ALS mice are compared to WT. There was no significant difference in glutamate concentrations over disease progression (Figure 1A). Glutamate concentrations were also not significantly increased from WT concentrations at any disease stage. GLT-1 and GluR1 levels decrease over disease progression. At end-stage, GLT-1 levels were 23% lower than end-stage WT values (*p* = 0.008) (Figure 1B). GluR1 concentrations at end-stage were 21% decreased from pre-onset levels (*p* = 0.035) (Figure 1C). There was no significant difference in GluR2 concentrations over disease progression (Figure 1C).

### GABA

**Figure 2:**
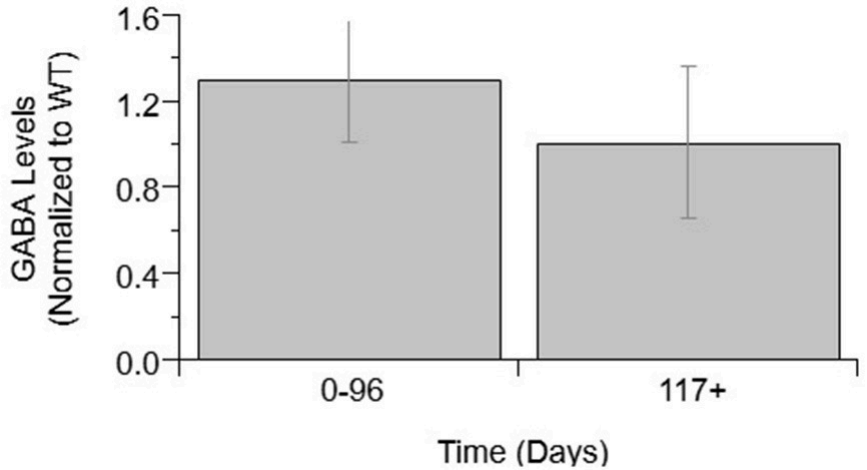
GABA levels over ALS disease progression. GABA level average normalized to wild-type (ratio presented as transgenic/WT) in two temporal stages: 0–96 days and 117+ days. Error bars represent SD.

Next, GABA levels were compared in ALS mice and WT mice (Figure 2). GABA concentrations showed no significant difference between WT and ALS transgenic values. Moreover, there was no significant change in GABA over the course of ALS disease stage or progression.

### Other Neuromodulatory Factors

**Figure 3:**
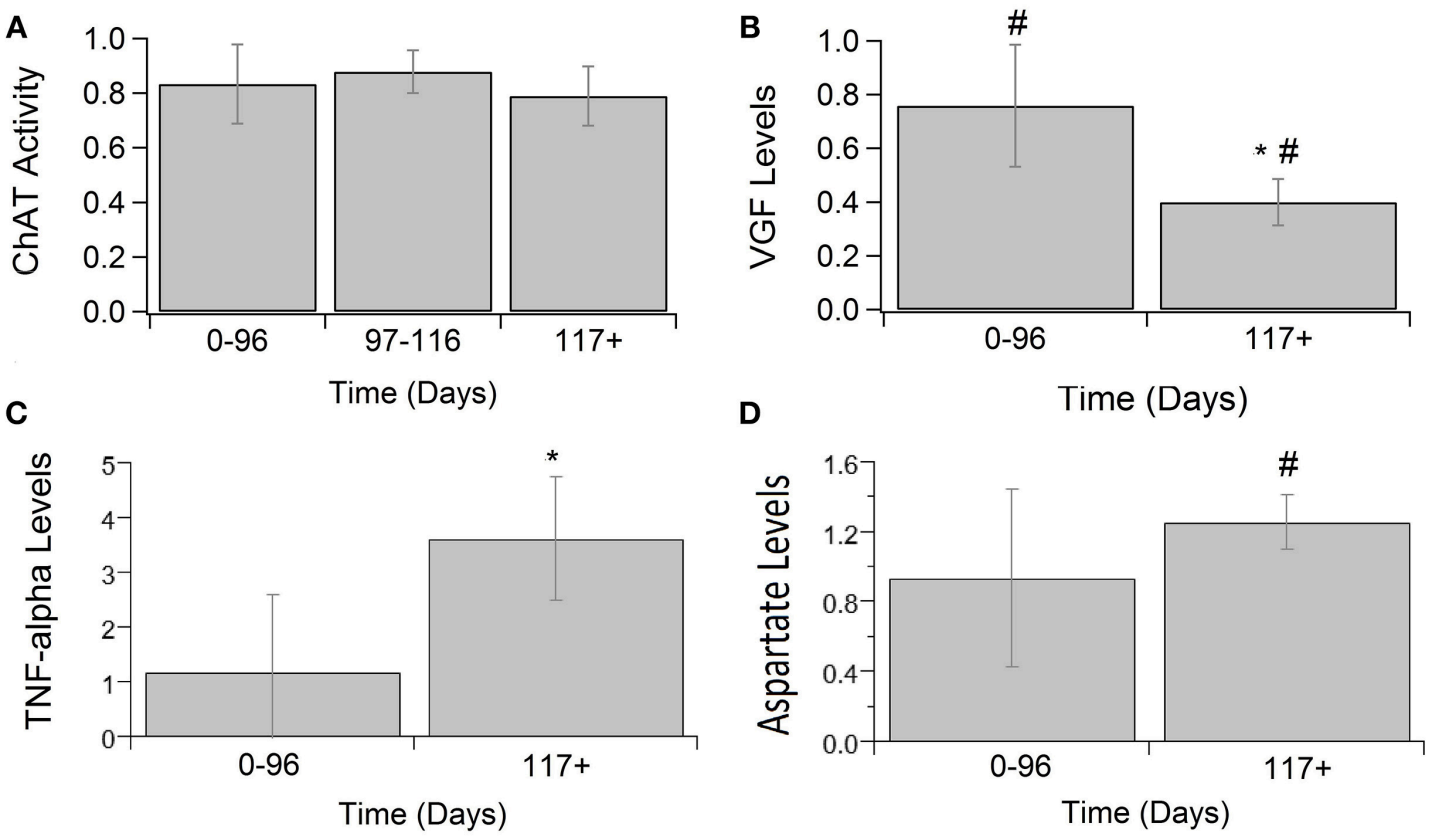
Calcium-related cytokine levels over ALS disease progression. ChAT activity **(A)**, VGF **(B)**, TNFα **(C)**, and aspartate **(D)** averages in SOD1-G93A normalized to wild type (ratio presented as SOD1-G93A/WT) in three stages: 0–96 days, 97–116 days, and 117 + days. SOD1-G93A VGF levels were significantly decreased compared to WT at 0–96 days (#*p* = 0.0036) and 117+ days (#*p* < 0.0001). SOD1-G93A VGF levels at end-stage are also significantly lower than early-stage (^*^p = 0.0002). TNFα (^*^p = 0.0488) and aspartate (#*p* = 0.0350) levels were significantly increased at end-stage compared to WT. Error bars represent SD.

Other calcium-related cytokines and neuromodulatory factors were examined over temporal SOD1-G93A disease progression (Figure 3). Choline acetyltransferase (ChAT), a transferase enzyme, is responsible for producing the neurotransmitter, acetylcholine (ACH). ChAT activity did not significantly vary over disease progression (Figure 3A). The lack of a significant trend in ChAT, which otherwise qualitatively appears to be depressed in SOD1-G93A compared to WT, is confounded by the large variance in experimental sample population. Nerve growth factor (VGF) is a secreted protein and neuropeptide precursor that may play a role in regulating energy homeostasis, metabolism and synaptic plasticity. VGF levels are depressed in SOD1-G93A ALS mixed cultures. VGF levels are significantly decreased from WT levels at both pre-onset (*p* = 0.0036) and end-stage (*p* < 0.0001), with end-stage being further significantly decreased from pre-onset (*p* = 0.0002; Figure 3B). Tumor necrosis factor alpha (TNFα) is a cytokine involved in inflammation, apoptosis, and synaptic function. TNFα expression in ALS mixed cultures significantly increased 362% from WT at end-stage (Figure 3C). Aspartate concentrations also significantly increased 25% from WT (Figure 3D).

### Glutamate, GABA, and Cytokine Relationships

**Figure 4:**
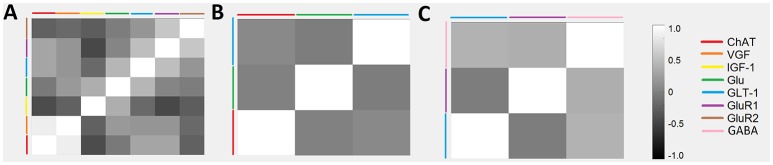
Temporal relationships between non-inflammatory regulators of astrocytes visualized as a cross-correlation matrix. Average concentrations of glutamate, GABA, and related cytokines for ALS mice were normalized to wild-type mice with data aggregated across three temporal stages: **(A)** 0–96 days, **(B)** 97–116 days, and **(C)** 117+ days. Highly positive correlations were found between VGF and ChAT at 0–96 days. Note that only factors with sufficient sample sizes (calculated using standard statistical power analysis) were included.

**Figure 5:**
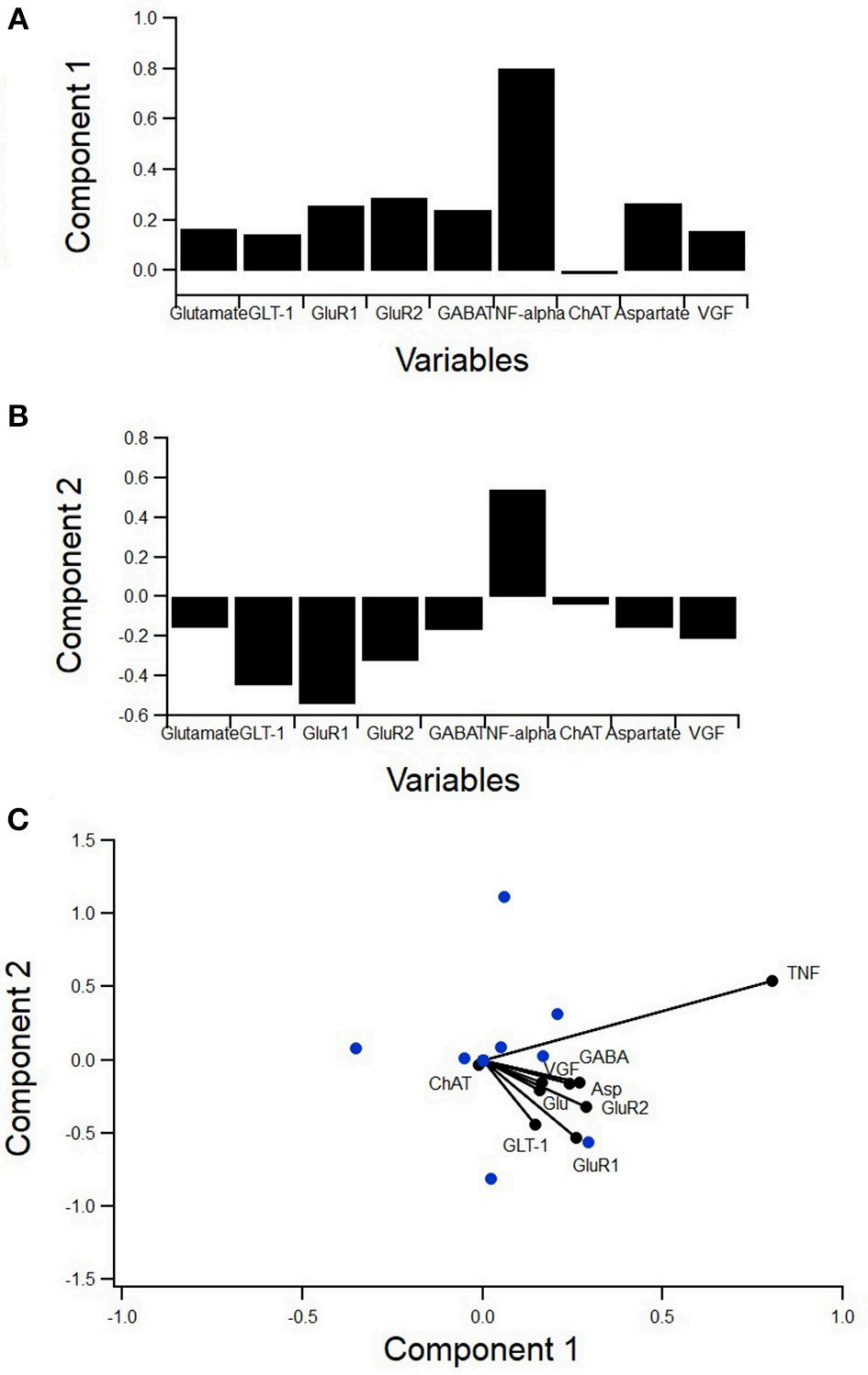
PCA to determine variance contribution of non-inflammatory regulators of astrocytes. Biplot showing variable contributions to the first two PCA-determined components **(A)** broken down to the first two components and the comprising variables **(B,C)**. Component 1 accounted for 55% of the variance and was almost completely comprised of TNFα **(B)**. Component 2 accounted for 17% of the variance and was mostly comprised of GABA and aspartate **(C)**.

A series of cross-correlation matrices were used to examine the correlations between various neuromodulatory regulators examined in this study (Figure 4). A cross-correlation matrix with correlations was constructed for each disease stage: 0–96 days (pre-onset); 97–116 days (post-onset); and 117+ days (end-stage). Highly negatively correlated factors are closer to −1, uncorrelated factors are closer to 0, and highly positively correlated factors are closer to 1; the illustrated gray scale represents the degree and sign of corresponding correlation. Only factors with a sample size calculated as having sufficient statistical power were included in each disease stage matrix. Thus, not every matrix included all factors. The pre-onset matrix included the most factors. The notable result from this analysis is that ChAT and VGF levels are high positively correlated at pre-onset (*p* = 0.0003; Figure 5).

A principal component analysis (PCA) was used to determine the individual components, which contribute the most to variance in the data (Figure 5). PCA is a type of exploratory and dimensionality reduction analysis. It is mathematically defined as an orthogonal linear transformation that transforms the data to a new coordinate system such that the greatest variance by some projection of the data comes to lie on the first coordinate (called the first principal component), the second greatest variance on the second coordinate, etc. The biplot displays how much the variables contribute to the variance of the first two principal components while also identifying reduced variable clusters (Figure 5A). The length of each factor's vector corresponds to the variance explained. Component 1 accounted for 55% of the variance and was almost completely comprised of TNFα (Figure 5B). Component 2 accounted for 17% of the variance and was mostly comprised of GABA and aspartate (Figure 5C). Interestingly, TNFα was nearly orthogonal to GluR1, indicating a lack of relationship.

**Figure 6:**
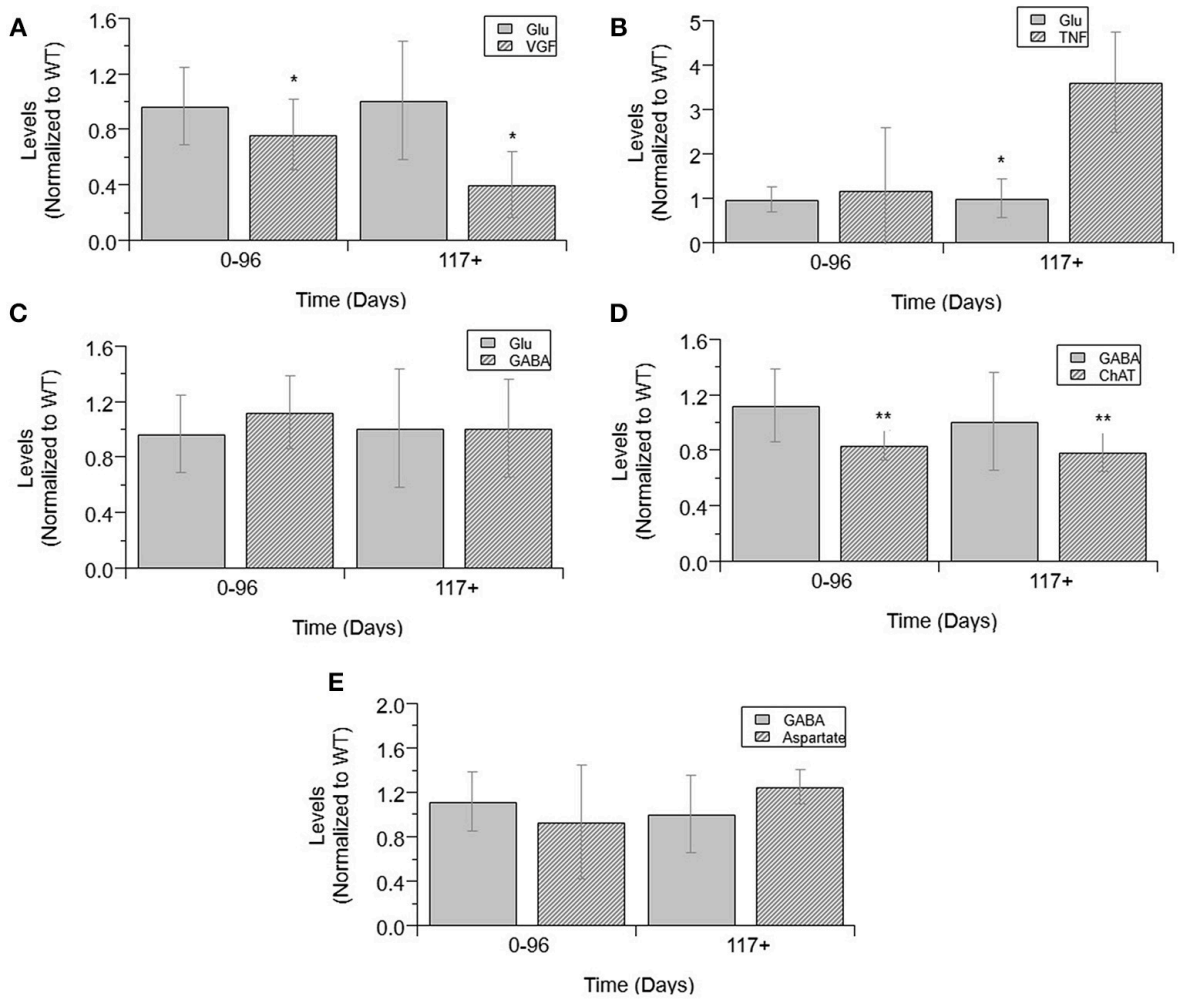
Cytokine levels compared to glutamate and GABA levels in mixed cultures over ALS disease progression. VGF **(A)**, TNFα **(B)**, GABA **(C)**, and glutamate **(D)** averages normalized to wild-type (ratio presented as transgenic/WT) in three temporal stages: 0-96 days, 97-116 days, and 117+ days. ChAT activity **(D)**, aspartate **(E)**, and GABA average normalized to wild-type (ratio presented as transgenic/WT) in two temporal stages: 0–96 days and 117+ days, as insufficient data was available for 97–116 days. VGF levels were significantly decreased from glutamate levels at both stages (^*^p < 0.05). TNFα levels were significantly increased from glutamate levels at 117+ days (^*^p = 0.0018). GABA levels were significantly decreased from ChAT levels at 0–96 days (^**^p = 0.0033) and at 117+ days (^**^p = 0.009). Error bars represent SD.

Finally, an analysis was performed to determine which normalized cytokine co-factor levels were significantly different than either glutamate or GABA in mixed cultures across each temporal disease stage (Figure 6). VGF levels were significantly decreased from glutamate at pre-onset and end-stage (*p* = 0.0331 and *p* = 0.0028; Figure 6A). TNFα was significantly increased from glutamate levels at pre-onset (*p* = 0.0018; Figure 6B). GABA levels were not significantly increased from glutamate at any ALS disease stage (Figure 6C). ChAT activity was significantly lower than GABA at pre-onset (*p* = 0.0033) and end-stage (*p* = 0.009; Figure 6D). Aspartate concentrations were not significantly lower than GABA at any disease stage (Figure 6E).

## Discussion

The metadata analysis results do not paint a picture of widespread, overt glutamate-mediated excitotoxicity in preclinical ALS as measured in mixed cell cultures. The key neuromodulators, namely glutamate, glutamate receptors, and transports, GABA, and ChAT, showed no so significant changes in ALS compared to WT or significant changes over the course of ALS disease progression. GLT-1 and GluR1 were significantly decreased at end stage, whereas aspartate was significantly increased at end stage. TNFα was significantly and drastically increased throughout ALS disease duration, whereas VGF was significantly decreased throughout the ALS disease course. Other than TNFα, directly measured changes in neuromodulators are modest at best. Below we discuss what these results suggest about the role of astrocyte-mediated neuromodulatory regulation in ALS etiology and potential treatments.

### Evaluating the Case for and Against ALS Excitotoxicity

Astrocytes play a critical role in reabsorbing excess extracellular glutamate and maintaining homeostasis for the motoneurons, which normally prevents excitotoxicity. There has been ongoing debate as to the presence or source of possible excitotoxicity (e.g., Leroy and Zytnicki, 2015; Rosenblum and Trotti, 2017; Martínez-Silva et al., 2018), especially given some animal model experiments suggest ALS motoneurons are hyperexcitable (Jiang et al., 2017) whereas other work suggests they are hypoexcitable (Martínez-Silva et al., 2018).

Previous clinical work illustrated evidence of increased glutamate levels in the CNS of ALS patients (Rothstein et al., 1990). Lack of resorption results in glutamate being left in the extracellular fluid resulting in continuous over-stimulation of the postsynaptic neurons (Rosenblum and Trotti, 2017). However, there was no identified increase in the excitatory transmitter, glutamate, in the preclinical ALS model mixed cell cultures examined in this study. As improved technology enables further examination, future experimental studies should focus on the specific localization of neurotransmitters to determine if key neurotransmitter changes are a function of spatial resolution, specific cell type, or a function of the type of ALS model.

Astrocytes use the enzyme glutamine synthetase (GS) to convert glutamate to glutamine as part of the glutamate/GABA-glutamine cycle (Norenberg and Martinez-hernandez, 1979; Bak et al., 2006). It is possible the constant levels of glutamate indicate SOD1-G93A astrocytes are reuptaking glutamate at higher rates and immediately converting it to maintain homeostasis. However, if glutamate was being taken in at higher rates it would require significant increases in GLT-1 and GluR to transport it into the cells. No such increases were found in this analysis. Rather, GLT-1 and GluR1 levels actually decreased compared to WT near end stage.

There are a few possible explanations for the decrease in GLT-1 and/or GluR1 levels. First, GLT-1 decreases could possibly be attributed to the difference in metabolic needs of astrocytes and neurons. Since GLT-1 activity in astrocytes leads to an influx of *Ca2*+ through the energy powered Na+-*Ca2*+ exchanger (Bazargani and Attwell, 2016), metabolic impairment in astrocytes would also lead to decreased GLT-1 activity. A second explanation for the decrease of GLT-1 and GluR1 over ALS disease progression could simply be due to the lower number of living or fully functioning cells in later stages of ALS. Finally, *if* there are potential cell-specific or spatially localized increases in excitation [spatial differences were not able to be examined in the present work], decreases in GLT-1 could suggest failed regulation or an inadequate compensatory response.

Beyond glutamate-related findings, there were several other neuromodulatory findings in the present study that provide further insight. For example, GABA, an inhibitory neurotransmitter, is not upregulated over the course of ALS in mixed cell cultures. If glutamate and other excitatory transmitters were in excess (either due to over-production or do to impaired re-uptake), it would be expected that GABA follow a similar trend due to innate compensatory mechanisms to balance excitation and inhibition. In the present study, neither GABA nor glutamate were significantly different in preclinical ALS compared to WT. Notably, aspartate, another excitatory neurotransmitter similar to glutamate, did show a slight increase in end stage preclinical ALS. However, ChAT, which is used to make the neurotransmitter acetylcholine, showed no significant difference.

In summary, there were no major changes in key neuromodulators that would point to very obvious glutamate-related excitotoxicity. Rather, the evidence presented here for mixed ALS cell cultures would suggest that direct neuromodulatory changes are subtle to modest, at least in SOD1 G93A ALS mixed cell cultures. This would suggest that, if excitotoxicity is present, it could be due to the fact that ALS neurons and astrocytes have properties that make them simply more susceptible or sensitive to even small changes in neuromodulation. Thus, even small or seemingly insignificant oscillatory increases or decreases in neuromodulators, which could easily be rectified with compensatory regulation in normal cells, could potentially wreak havoc in ALS cells. In fact, as discussed in more detail in the Section Astrocyte GluR as a Potential Pre-onset ALS Treatment Target, motoneurons are mathematically and functionally more susceptible to such instability (Mitchell and Lee, 2012; Irvin et al., 2015). Other recent work has also illustrated that motoneuron microcircuits are more prone to homeostatic dysregulation (Brownstone and Lancelin, 2018). Given the large variance in experimental data, small changes that were statistically insignificant may actually be physiologically significant to ALS cells. If ALS cells are not able to sufficiently control their microenvironment, there could be spatial “pockets” or temporal phases of both hyperexcitability and hypoexcitability. This could mean that both hyperexcitability and hypoexcitability could co-exist in ALS, which could explain the discrepancies seen in previous literature.

### Astrocyte GLT-1 as a Potential Post-onset ALS Treatment Target

Due to the modest success of Riluzole, a drug used to treat ALS that is thought to increase glutamate reuptake, there has been significant focus on finding ways to increase the reuptake of glutamate by astrocytes (Do-Ha et al., 2017). Additionally, post-mortem studies of ALS tissue and mice have found reduced levels of EAAT2 and GLT-1, respectively (Rothstein et al., 1995; Pardo et al., 2006). It is believed that decreased GLT-1 levels are contributing to the accumulation of extracellular glutamate and increasing the levels could help prevent the hyperexcitability of the motoneurons. Few experiments have had any notable success, however, due to the complex interaction and timing of the pathophysiology (Li et al., 2015). The present study found that GLT-1 levels only decrease significantly from post-onset to end-stage (Figure 1B). This finding suggests that increasing GLT-1 levels before ALS end-stage may increase glutamate reuptake, thereby prolonging survival or minimally prolonging muscle function after ALS onset.

If cell-specific or spatially localized excitation is present, extrinsically increasing GLT-1 levels before ALS end-stage may increase glutamate reuptake, thereby prolonging survival or minimally prolonging muscle function after ALS onset. Recent analysis has illustrated the potential impact of treatments that can increase patient quality of life by preserving muscle function for as long as possible even if corresponding survival increases are not dramatic. For example, similar metadata analysis of oxidative stress treatments in high copy SOD1-G93A ALS mice prolong limb muscle function by 59.6% but only prolong survival by 11.2% (Bond et al., 2018).

### Astrocyte GluR as a Potential Pre-onset ALS Treatment Target

The influx of *Ca2*+ caused by the overstimulation of motoneurons leads to cell death as *Ca2*+ is involved in many apoptotic pathways. The permeability of glutamate receptors is determined by GluR subunits. Glutamate receptors with GluR1 subunits are highly permeable to *Ca2*+ and causes the mobilization of intracellular *Ca2*+ when activated (D'antoni et al., 2011). GluR2 lacking glutamate receptors are highly permeable to *Ca2*+ (Grosskreutz et al., 2010). Studies have shown that a lack of GluR2 increased motoneuron degeneration (Van Damme et al., 2005) in mice and increasing GluR2 levels in motoneurons prolonged survival of mice (Tateno et al., 2004). While decreased GluR2 levels were not observed in ALS mixed cultures in this study, there was a decrease in GluR1 levels at end-stage when compared to post-onset (Figure 1C), possibly from the high levels of cell death that have occurred by that point. Additionally, GluR1 is elevated from WT at post-onset, though this difference was not found to be significant, likely owing to small sample size. These findings suggest that reducing GluR1 levels in astrocytes at pre-onset, prior to their elevation post-onset, could reduce intracellular *Ca2*+ levels and *Ca2*+ dysregulation effects. Due to the limited number of data points available for intracellular *Ca2*+ measured, the relationships of *Ca2*+ and the GluR subunits were unable to be examined.

Of course, treatment at “pre-onset” in clinical terms would mean treatment in familial or genetic cases of ALS, referred to as FALS, where disease predisposition or the presence or pre-symptomatic ALS could be identified well before symptoms. Such a treatment is not realistic at this point because precise, reliable tests for explicit ALS identification [prior to symptom onset] are not yet available, even for FALS. The ability to identify more reliable biomarkers, genetic or otherwise, that predict future onset of ALS would open up the possibility of prophylactic treatment pre-onset. Such an approach would be similar to the familial amyloid-β treatments being tested prophylactically in a known Colombian family where genetic tests can predict with certainty who will get a very specific form of familial Alzheimer's Disease well before cognitive symptoms appear (Reardon, 2018). Early preclinical evidence illustrates that infamous amyloid-β treatments, when used alone, may require prophylactic treatment to be successful (Huber et al., 2018). While many FALS markers have been identified, albeit SOD1, C9orf72, or the even more rare mutations like FUS/TLS, VAPB, etc. (Ajroud-Driss and Siddique, 2015), no such marker has yet been identified that definitively, with great sensitivity and specificity, can pre-determine a future FALS onset.

### Elucidating Etiology of *Ca2+* Increase in ALS

Measuring intracellular calcium can be very complicated. *Ca2*+ transients are spatially localized within the astrocyte (Bazargani and Attwell, 2016). Due to methodological constraints, the full intracellular *Ca2*+ store may not be found by simply looking at the soma, which complicates experimental *Ca2*+ examination in astrocytes at the present time. Given the complications of local, spatial, and temporal *Ca2*+ experimental examination, it is difficult to determine what mechanism is most directly related to the increases in *Ca2*+ that have been previously identified in ALS (Irvin et al., 2015).

In healthy astrocytes, glutamate transporter activity leads to an increased entry of *Na*+ as GLT-1 couples glutamate and *Na*+ (Bazargani and Attwell, 2016). In addition to the *Na*+*-K*+ ATPase pump, the *Na*+*-Ca2*+ exchange provides a way for this *Na*+ to exit while *Ca2*+ enters. Therefore, an increase in glutamate uptake through GLT-1 activity can lead to increase in intracellular *Ca2*+. ALS astrocytes do not appear to increase glutamate uptake over disease progression and, consequently, do not increase intracellular *Ca2*+ concentrations. Neurons, however, can become increasingly more susceptible to glutamate excitotoxicity over time and, thus, see a rise in intracellular *Ca2*+.

Interestingly, early pre-onset elevation of intracellular *Ca2*+ in SOD1-G93A mixed cell cultures occurs without a concurrent increase in intracellular glutamate. Thus, the mechanisms causing failed astrocytic *Ca2*+ regulation is not explicitly tied to glutamate. Exocytosis of GABA is, in part, dependent on the influx of *Ca2*+ through voltage-gated calcium channels (VGCCs) (Sitges and Chiu, 1995). However, we found no increase in GABA in ALS preclinical model mixed cell cultures. Thus, unless there are localized changes in excitatory neurotransmitters that have not yet been experimentally measured, the increase in *Ca2*+ appears to not be directly tied to changes in excitatory neurotransmitter balance—at least not in preclinical ALS mixed cell cultures analyzed in the present study. Interestingly, a recent study showed that clinical patients with the C9ORF72 mutation had motoneurons that were more vulnerable to *Ca2*+ permeable AMPA receptors (Selvaraj et al., 2018).

*Ca2*+ increases could come from a variety of mechanisms beyond direct or significant changes in neuromodulatory transmission. As proposed in prior work (Mitchell and Lee, 2012; Mitchell et al., 2015b; Hollinger et al., 2016; Kim et al., 2016), failed homeostasis in nearly ten different major pathways could explain how a variety of different perturbations that result in ALS and identified homeostatic regulatory instability. *Ca2*+ regulation is also greatly impacted by changes in energetic pathways (namely mitochondria and endoplasmic reticulum *Ca2*+ stores) and oxidative stress (Bond et al., 2018), and to a lesser degree, by apoptotic signaling (Irvin et al., 2015).

Motoneurons are more prone to instability due to their long length (Mitchell and Lee, 2012), which requires transport and signaling over very long distances in the axon. Any delays or offset in signaling within cells or even between cells, such as between neurons and astrocytes, could contribute to oscillatory instability. The oscillations seen in CNS experimental data, and especially *Ca2*+, support the homeostatic instability theory (Irvin et al., 2015) of ALS. Given the very small margin of error for homeostatic regulation in motoneurons (Mitchell and Lee, 2012), localized examination of *Ca2*+ with sufficient spatio-temporal resolution in the future could provide key evidence for experimentally confirming the contributions, or lack thereof, of neuromodulatory regulation mechanisms in neurons and astrocytes.

### Puzzling Non-correlation Between Calcium-Permeable GluR1 and TNFα

In general, TNFα production and secretion is believed to be highly dependent upon calcium levels (Watanabe et al., 1996). One mechanism proposed for TNFα-induced neuron death is through the rapid TNFα-induced surface expression changes of AMPA-type glutamate receptors, such as GluR1 (Ferguson et al., 2008). Dysregulation of glutamate receptor trafficking can alter neuronal calcium permeability and contribute to excitotoxic vulnerability (Olmos and Llado, 2014; Kia et al., 2018).

Therefore, the lack of a statistically significant relationship between TNFα and GluR1 levels in SOD1-G93A mixed cultures was unexpected (Figure 6). Moreover, the PCA (Figure 5A) showed TNFα and GluR1 levels to be practically decoupled given TNFα is almost orthogonal to GluR1 on the biplot (Figure 5A), which also indicates a lack of relationship.

Of course, TNFα has a complex relationship with many cellular processes. It could be that the data variance identified and imparted by TNFα in the PCA is more related to its other roles, including inflammation, synaptic function, and especially caspase-8 initiated apoptosis (Kia et al., 2018). On a more detailed level, TNFα and NF-κB have been shown to participate in oscillatory positive feedback loops wherein NF-κB can regulate TNFα transcription. This process plays a key role in regulating inflammatory responses across a variety of cell types, and has been found to be central in the FUS mutation of ALS (Pekalski et al., 2013) as well as other neurodegenerative diseases. Another possible role of TNFα could be its relationship with glial derived neurotrophic factor, GDNF. Recent work has shown that TNFα contributes to rises in astrocytic GDNF that have a protective effect on neuron damage (Brambilla et al., 2016); in fact, the TNFα –GDNF mechanism is currently being sought as a potential therapeutic target. Most recently, astrocytes in the ALS FUS model were shown to induce motoneuron death directly via the release of TNFα (Kia et al., 2018).

Thus, while more research is necessary to examine the puzzling lack of relationship seen between TNFα and GluR1 levels, it appears both GluR1 and TNFα are each having an impact on ALS pathology, but the direct relationship between TNFα and GluR1 is simply not substantial in SOD1-G93A ALS mixed cell cultures.

### Aspartate Increases Near End-Stage

Aspartate is another excitatory neurotransmitter, similar to glutamate, that is found in the brain. In the present metadata analysis of preclinical ALS mixed cell cultures, the aspartate increase is not seen until end-stage. This supports the finding of normal or slightly decreased aspartate levels in cerebral spinal fluid serum of ALS patients with a mild disease course (Niebroj-Dobosz and Janik, 1999). In severely progressing patients, aspartate levels were increased, and GABA levels were normal or increased. When the imbalance of these amino acids favors the excitatory over the inhibitory, it appears to contribute to a more rapid decline in survival.

### Failed Regulation Could Contribute to Spatial Spread of ALS

An important characteristic of ALS is the spread of motoneuron degeneration and corresponding cell death. Recent research has found that transplanting familial ALS astrocytes into healthy mice causes ALS-like degeneration in non-specific healthy neurons (Qian et al., 2017). The spread has been partially attributed to a prion-like mechanism (Grad et al., 2015). More research is needed to better understand the mechanistic etiology of the spread of ALS. Nonetheless, one of the most prominent theories has been related to excitotoxic spillover. For example, gliotransmitters, such as increased glutamate or decreased GABA, can propagate the rise of intracellular calcium levels in astrocytes and adjacent neurons. Glutamate can be released from astrocytes through *Ca2*+-activated bestrophin-1 anion channels. Changes in intracellular calcium levels in astrocytes can also affect the activity of membrane transporters, such as GluRs (Bazargani and Attwell, 2016). Yet, in the present metadata analysis in preclinical ALS mixed cell cultures, we did not see glaring evidence of severe excitotoxic spillover in preclinical ALS mixed cell cultures, as key excitatory (glutamate) and inhibitory transmitters (GABA) were not elevated. However, the identified decrease in inhibitory GLT-1 and increase of excitatory aspartate that occurs with disease progression does suggest there is at least a mild form of neuromodulatory regulation, or lack thereof, that is contributing to ALS etiology. Additionally, the presented analysis does illustrate potential evidence of upregulation, spillover or spread of other chemokines, especially TNFα. Dysregulated or delayed neuromodulator regulatory signaling between neurons and astrocytes should be further explored in future experimental work; evidence of dysregulation could be subtler than what is typically seen with primary excitotoxicity but could nonetheless help to explain the underlying “spread” mechanism(s) of ALS.

### Other Non-inflammatory Functions of Astrocytes

ALS-causing genes are also linked to lipid homeostasis, glucose homeostasis, mitochondrial formation, ATP production, and other metabolic functions (Ngo and Steyn, 2015). Extracellular concentrations of ATP in the CNS regulate activation and migration of immune and glial cells and increase in response to trauma and inflammation (Gandelman et al., 2010). In astrocytes, it can cause pro-inflammatory signaling which causes an increased production of nitric oxide and various chemokines (Gandelman et al., 2010). It has been found that G93A astrocytes degrade ATP at a faster rate than non-transgenic astrocytes and display ATP-dependent proliferation. It has also been shown that high extracellular concentrations of ATP can cause non-transgenic astrocytes to induce motoneuron death (Gandelman et al., 2010). Evidence shows that a complicated cycle of bioenergetic deficits may worsen disease progression over time (Ngo and Steyn, 2015). An analysis of extracellular ATP was not completed as part of this study due to lack of data meeting inclusion criteria. However, a similar analysis of ATP in the CNS shows deficiency throughout the SOD1-G93A ALS life span (Irvin et al., 2015). Like glutamate, the temporal trends of metabolic homeostasis need to be determined to identify the most effective time for potential therapeutic intervention.

## Limitations

The biggest limitations to any metadata analysis is the ability to aggregate data sources in a way that sufficiently increases sample size but not so aggregated that group definitions are too diverse to provide meaningful insight. The adage of “apples to apples” and “apples to oranges” is a good analogy for metadata analysis. To carry this analogy into an easy to understand example, a specific metadata analysis group defined as “apples” must literally include all apples, although different individual apples in the group may look slightly different (red, green, yellow, etc.). Transitioning to the present work, “glutamate,” for example, include metrics that are slightly different: acutely released glutamate (Milanese et al., 2011; Albano et al., 2013; etc.), steady state extracellular measurements of glutamate (Alexander et al., 2000), whole brain MRI levels of glutamate (Niessen et al., 2007; Choi et al., 2009, etc.), among many other slight variations of direct or indirect glutamate measurement, which are contained within the “glutamate” data aggregate as shown in Table 2 and the Supplementary Data Sheet. It would be preferable to have more specifically defined groups based on different glutamate locations, functions, and/or experimental methodology. However, the number of available studies and their corresponding heterogeneity does not allow more specifically defined sub-groups at the time of this writing; there simply would not be enough statistical power. Thus, the present work utilized more generalized groupings and aggregation schemes (see Methods) that met the standardized criteria and statistical power required by metadata analysis. Nonetheless, this study's presented results still provide new high-level insights as to which of the different biomedical concepts are impacting astrocyte-mediated ALS neuromodulatory regulation over the course of ALS disease progression. Such high-level insight helps researchers to better synthesize complex interactions as well as assess and prioritize biomedical concept research aimed at improving diagnostics, prognostics, and therapeutics. It is an acknowledged limitation that many more data sources are needed to construct narrow aggregate definitions that enable very specific mechanistic hypotheses to be confirmed.

## Author Contributions

KJ: data collection, statistical analysis, results interpretation, drafting of initial manuscript, critical review of content; JM: data collection, results interpretation, critical review of content; AS: data collection, statistical analysis, critical review of content; CM: framing of study, project oversight, results interpretation, drafting of final manuscript, critical review of content.

### Conflict of Interest Statement

The authors declare that the research was conducted in the absence of any commercial or financial relationships that could be construed as a potential conflict of interest.
