# Supplementary material for: Astrocyte-Mediated Neuromodulatory Regulation in Preclinical ALS: A Metadata Analysis
Source: Front Cell Neurosci. 2018 Dec 17;12:491. doi: 10.3389/fncel.2018.00491 (PMC6305074; doi:10.3389/fncel.2018.00491)
Supplement: Supplementary file 1 [file Data_Sheet_1.PDF]

Supplementary Data Sheet

Astrocyte-mediated neuromodulatory regulation in preclinical ALS: a metadata analysis

Front. Cell. Neurosci. (2018)

Kathleen Jordan, Joseph Murphy, Anjanya Singh, and Cassie S. Mitchell

<u>Contains:</u>

SDS Figure 1

SDS Figure 2

SDS Table 1

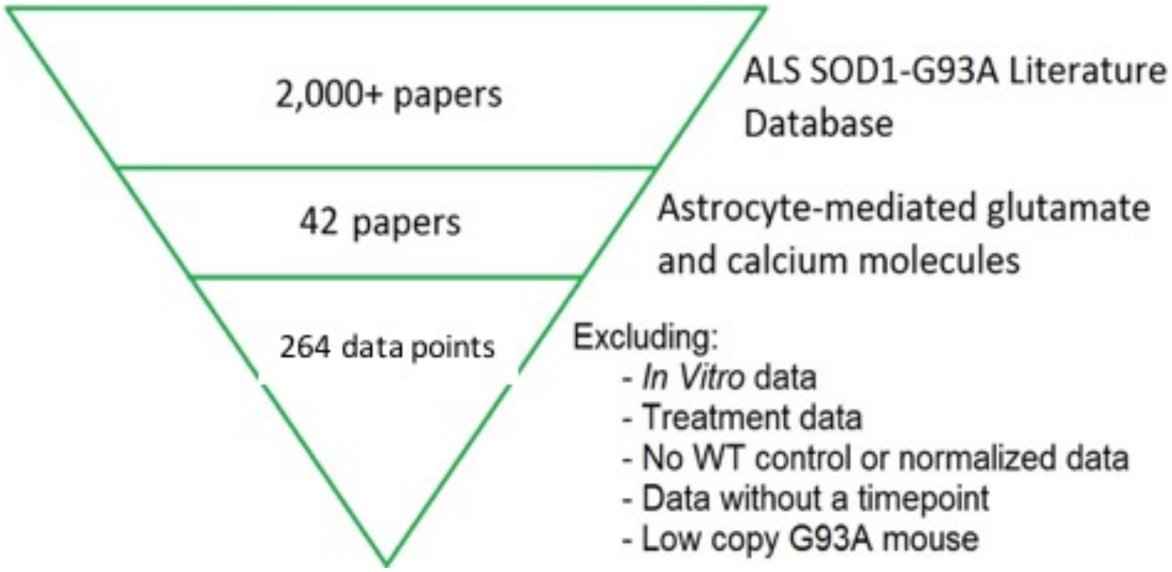

SDS Figure 1. Data selection process. A literature database of over 2,000 published articles on ALS SOD1-G93A mouse models was reduced to 42 peer-reviewed articles using search terms and inclusion/exclusion criteria. A total of 264 normalized data points were quantified from these papers and used in the study's analysis. The main article lists more details on search terms (Tables 1, 2) and data recapture/aggregation protocols (see Methods).

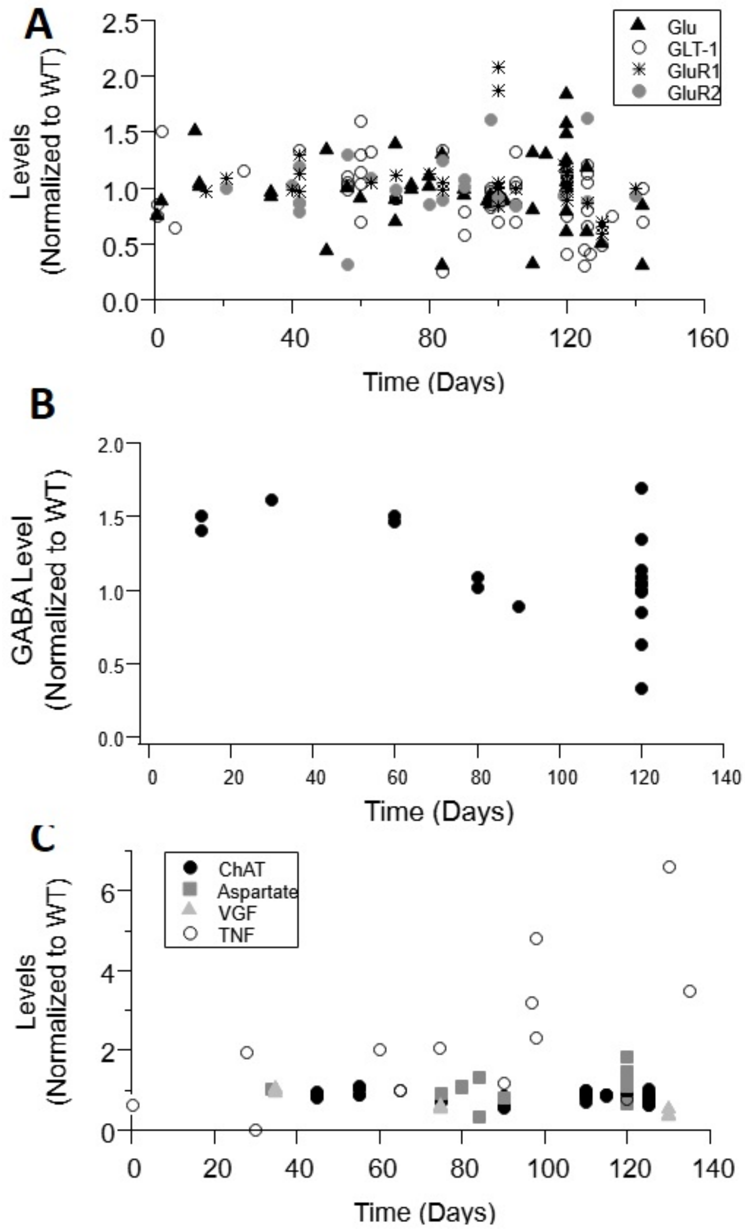

SDS Figure 2. Non-aggregated data included in the analysis. data included measures of intracellular glutamate, GTL-1, GluR1, GluR2 (A), GABA (B), ChAT activity, Aspartate, VGF, and TNF-alpha (C). Only studies that presented quantified data for both transgenic and age-matched wild type control mice were included. A total of 42 articles with quantifiable experimental data had data extracted for analysis. Quantified data was used to construct ratios of transgenic to wild type (transgenic / WT) for each included metric at each temporal disease stage.

SDS Table 1. Full list of included peer-reviewed data sources with details on data source and aggregation. The original data source citation information is given in the following fields: “PMID” (PubMed ID number), “Author”, “Journal”, “Pages”, “Volume”, “Issue”, and “Year”. “Category” describes what type of data was taken from each paper and where its data was used for aggregation in the present study. “Gene Cross” lists the genetic backcrossing of the ALS model; when stated in the original data source, most studies used SOD1-G93A mice with B6SJL or C57/BL genetic backgrounds. “Mouse type” lists the type of original ALS data and the controls used, either wildtype (WT) or non-transgenic (NT) age-matched mice. “Figure” refers to the original data source’s figure number or table from which data was recaptured. “Methods” summaries the original data source’s measurement procedure and briefly describes how it obtained the data..

PMID	Author	Journal	Pages	Title	Vol	Iss	Year	Category	Gene Cross	Mouse Type	Figure	Methods
18432310	Zhao Z, Lange DJ, Ho L, Bonini S, Shao B, Salton SR, Thomas S, Pasinetti GM	Int J Med Sci	92-9	Vgf is a novel biomarker associated with muscle weakness in amyotrophic lateral sclerosis (ALS), with a potential role in disease pathogenesis.	5	2	2008	VGF		G93A Control	2 & 3	Expression analysis and immunohistochemistry on CSF, serum, and spinal cord sections
18951954	Martinez JA, Francis GJ, Liu WQ, Pradzinsky N, Fine J, Wilson M, Hanson LR, Frey WH 2nd, Zochodne D, Gordon T, Toth C.	Neuroscience.	908-25	Intranasal delivery of insulin and a nitric oxide synthase inhibitor in an experimental model of amyotrophic lateral sclerosis	157	4	2008	GluR1	B6SJL	G93A Control	8	qRT-PCR of spinal cord sections
10724110	Guo Z, Kindy MS, Kruman I, Mattson MP	J Cereb Blood Flow Metab	463-8	ALS-linked Cu/Zn-SOD mutation impairs cerebral synaptic glucose and glutamate transport and exacerbates ischemic brain injury.	20	3	2000	Glutamate		G93A Control	2	Synaptosomes prepared from cerebral hemispheres
10737625	Alexander GM, Deitch JS, Seeburger JL, Del Valle L, Heiman-Patterson TD	J Neuro-chem	1666-73	Elevated cortical extracellular fluid glutamate in transgenic mice expressing human mutant (G93A) Cu/Zn superoxide dismutase.	74	4	2000	Glutamate,	B6SJL	G93A Control	Table 1, Fig. 2, & Fig. 6	Intracerebral dialysis and quantification of amino acids using precolumn derivatization of brain sections
11723166	Bendotti C, Tortarolo M, Suchak SK, Calvaresi N, Carvelli L, Bastone A, Rizzi M, Rattray M, Mennini T	J Neuro-chem	737-46	Transgenic SOD1 G93A mice develop reduced GLT-1 in spinal cord without alterations in cerebrospinal fluid glutamate levels.	79	4	2001	Glutamate	C57BL/(	G93A Control	3, 5, 6, & 8	Mice were killed and blood and spinal cord smaples were collected
11790392	Deitch JS, Alexander GM, Del Valle L, Heiman-Patterson TD	J Neurol Sci	117-26	GLT-1 glutamate transporter levels are unchanged in mice expressing G93A human mutant SOD1.	193	2	2002	GLT-1	B6SJL	G93A Control	4	Mice were euthanized and cortex, brain stem, cervical and lumbar spinal cord sections were removed
12915461	Guo H, Lai L, Butchbach ME, Stockinger MP, Shan X, Bishop GA, Lin CL	Hum Mol Genet	2519-32	Increased expression of the glial glutamate transporter EAAT2 modulates excitotoxicity and delays the onset but not the outcome of ALS in mice.	12	19	2003	Glutamate		G93A Control	7	Immunostaining of lumbar spinal cord sections
15033338	Raiteri L, Stigliani S, Zappettini S, Mercuri NB, Raiteri M, Bonanno G	Neuro-pharmacology	782-92	Excessive and precocious glutamate release in a mouse model of amyotrophic lateral sclerosis.	46	6	2004	Glutamate	B6SJL	G93A Control	Table 2	Synaptosomes were prepared from spinal cords
15512905	Chen LC, Smith A, Ben Y, Zukic B, Ignacio S, Moore D, Lee N	Amyo-troph Lat Scler Oth Mot Neur Disord	164-71	Temporal gene expression patterns in G93A/SOD1 mouse.	5	3	2004	GLT-1	B6SJL	G93A Control	1	Gene expression in spinal cord tissue
15635412	Rothstein JD, Patel S, Regan MR, Haenggeli C, Huang YH, Bergles DE, Jin L, Dykes Hoberg M, Vidensky S, Chung DS, Toan SV, Bruijn LI, Su ZZ, Gupta P, Fisher PB	Nature	73-7	Beta-lactam antibiotics offer neuroprotection by increasing glutamate transporter expression.	433	7021	2005	GLT-1	B6SJL	G93A Control	4	Protein expression in spinal cord tissue

PMID	Author	Journal	Pages	Title	Vol	Iss	Year	Category	Gene Cross	Mouse Type	Figure	Methods
15737434	Crochemore C, Pena-Altamira E, Virgili M, Monti B, Contestabile A	Neuro-chem Int	357-68	Disease-related regressive alterations of forebrain cholinergic system in SOD1 mutant transgenic mice.	46	5	2005	ChAT	B6SJL	G93A Control	Table 2, Fig. 1, Fig. 3, & [illegible]	Animals were decapitated and various brain samples were removed
15852403	Kaspar BK, Frost LM, Christian L, Umapathi P, Gage FH	Ann Neurol	649-55	Synergy of insulin-like growth factor-1 and exercise in amyotrophic lateral sclerosis.	57	5	2005	IGF-1	B6SJL	G93A Control	3	qRT-PCR of lumbar spinal cord sections
16087196	Petri S, Schmalbach S, Grosskreutz J, Krampfl K, Grothe C, Dengler R, Van Den Bosch L, [illegible]	J Neurol Sci	25-30	The cellular mRNA expression of GABA and glutamate receptors in spinal motor neurons of SOD1 mice.	238	1-2	2005	GluR1 & Gl	B6SJL	G93A Control	1	Densitometeric analysis on spinal motor neurons
16309674	Spalloni A, Geracitano R, Berretta N, Sgobio C, Bernardi G, Mercuri NB, Longone P, Ammassari-Teule M	Exp Neurol	505-14	Molecular and synaptic changes in the hippocampus underlying superior spatial abilities in pre-symptomatic G93A+/+ mice overexpressing the human Cu/Zn superoxide dismutase (Gly93 --> ALA) mutation.	197	2	2006	GluR1 & Gl	B6SJL	G93A Control	Table 1, Fi.g 4, & Fig. 5	Quanititative PCR and Western Blotting of frontal cortex and hippocampus
16323214	Tortarolo M, Grignaschi G, Calvaresi N, Zennaro E, Spaltro G, Colovic M, Fracasso C, Guiso G, Elger B, Schneider H, Seilheimer B, Caccia S, Bendotti C	J Neurosci Res	134-46	Glutamate AMPA receptors change in motor neurons of SOD1G93A transgenic mice and their inhibition by a noncompetitive antagonist ameliorates the progression of amytrophic lateral sclerosis-like disease.	83	1	2006	GluR2	C57BL/(	G93A Control	2 & 5	Quanititative PCR and Western Blotting of spinal cord sections
16567804	Boston-Howes W, Gibb SL, Williams EO, Pasinelli P, Brown RH Jr, Trotti D	J Biol Chem	14076-8	Caspase-3 cleaves and inactivates the glutamate transporter EAAT2.	281	20	2006	GLT-1	B6SJL	G93A Control	7	Densitometeric analysis on spinal cord homogenates
16753145	Pardo AC, Wong V, Benson LM, Dykes M, Tanaka K, Rothstein JD, Maragakis NJ	Exp Neurol	120-30	Loss of the astrocyte glutamate transporter GLT1 modifies disease in SOD1(G93A) mice.	201	1	2006	GLT-1		G93A Control	1 & 2	Quantitative densitometry in lumbar spinal cord and cortex
16877542	Wu DC, Re DB, Nagai M, Ischiropoulos H, Przedborski S	Proc Natl Acad Sci U S A	12132-7	The inflammatory NADPH oxidase enzyme modulates motor neuron degeneration in amyotrophic lateral sclerosis mice.	103	32	2006	IGF-1	C57BL/(	G93A Control	4	Spinal cords were fixed and processed for immunostaining
17432958	Niessen HG, Debska-Vielhaber G, Sander K, Angenstein F, Ludolph AC, Hilfert L, Willker W, Leibfritz D, Heinze HJ, Kunz WS, Vielhaber [illegible]	Eur J Neurosci	1669-77	Metabolic progression markers of neurodegeneration in the transgenic G93A-SOD1 mouse model of amyotrophic lateral sclerosis.	25	6	2007	Glutamate	B6SJL	G93A Control	Table 2, Fig. 3, & Fig. 4	Mice were killed by decapitation and the CNS was then divided into brainstem, cerebellum, cortex and whole spinal cord in a brain medium
17597610	Messi ML, Clark HM, Prevette DM, Oppenheim RW, Delbono O	Exp Neurol	52-63	The lack of effect of specific overexpression of IGF-1 in the central nervous system or skeletal muscle on pathophysiology in the G93A SOD-1 mouse model of ALS.	207	1	2007	IGF-1	C57BL/(	G93A Control	8 & 9	Radioimmunoassay of various regions of the CNS

PMID	Author	Journal	Pages	Title	Vol	Iss	Year	Category	Gene Cross	Mouse Type	Figure	Methods
18279310	Zhao P, Ignacio S, Beattie EC, Abood ME	Eur J Neurosci	572-9	Altered presymptomatic AMPA and cannabinoid receptor trafficking in motor neurons of ALS model mice: implications for excitotoxicity.	27	3	2008	GluR1 & Gl	B6SJL	G93A Control	2, 4, & 5	Mice were euthanized and lumbar spinal cords were removed
19296491	Crochemore C, Virgili M, Bonamassa B, Canistro D, Pena-Altamira E, Paolini M, Contestabile A	Muscle Nerve	548-52	Long-term dietary administration of valproic acid does not affect, while retinoic acid decreases, the lifespan of G93A mice, a model for amyotrophic lateral sclerosis.	39	4	2009	ChAT	B6SJL	G93A Control	1	Mice were killed by decapitation and sections of the spinal cord and hippocampus were removed
19323997	Yang Y, Gozen O, Watkins A, Lorenzini I, Lepore A, Gao Y, Vidensky S, Brennan J, Poulsen D, Won Park J, Li Jeon N, Robinson MB, Rothstein JD	Neuron	880-94	Presynaptic regulation of astroglial excitatory neurotransmitter transporter GLT1.	61	6	2009	GLT-1	B6SJL	G93A Control	7	Cortical astrocyte cultures were prepared from mouse pups.
19930399	Choi JK, Kustermann E, Dedeoglu A, Jenkins BG	Eur J Neurosci	2143-50	Magnetic resonance spectroscopy of regional brain metabolite markers in FALS mice and the effects of dietary creatine supplementation.	30	11	2009	Glutamate	B6SJL	G93A Control	Table 1, Table 2, & Table 3	Mice were decapitated and sensorimotor cortex, cerebellum and medulla were dissected
20152807	Gu R, Hou X, Pang R, Li L, Chen F, Geng J, Xu Y, Zhang C	Biochem Biophys Res Commun	481-6	Human adipose-derived stem cells enhance the glutamate uptake function of GLT1 in SOD1(G93A)-bearing astrocytes.	393	3	2010	Glutamate	B6SJL	G93A Control	2	Protein expression and uptake in primary astrocytes
20333481	Yang X, Cheng B	J Mol Neurosci	145-53	Neuroprotective and anti-inflammatory activities of ketogenic diet on MPTP-induced neurotoxicity.	42	2	2010	TNF-alpha	C57BL/(	G93A Control	6	Mice were sacrificed and brains were dissected
20441996	Fang L, Teuchert M, Huber-Abel F, Schattauer D, Hendrich C, Dorst J, Zettlmeissel H, Wlaschek M, Scharffetter-Kochanek K, Kapfer T, Tumani H, Ludolph AC, Brettschneider J	J Neurol Sci	51-6	MMP-2 and MMP-9 are elevated in spinal cord and skin in a mouse model of ALS.	294	1-2	2010	TNF-alpha	B6SJL	G93A Control	Table 1	After cervical dislocation of mice, cerebellum and skin were dissected and snap-frozen in liquid nitrogen
20660618	Tsai KJ, Yang CH, Fang YH, Cho KH, Chien WL, Wang WT, Wu TW, Lin CP, Fu WM, Shen CK	J Exp Med	1661-73	Elevated expression of TDP-43 in the forebrain of mice is sufficient to cause neurological and pathological phenotypes mimicking FTLD-U.	207	8	2010	GABA		TG ALS Model Control	4	Immunostaining measurement of primary hippocampal neuron cultures
21175617	Milanese M, Zappettini S, Onofri F, Musazzi L, Tardito D, Bonifacino T, Messa M, Racagni G, Usai C, Benfenati F, Popoli M, Bonanno G.	J Neurochem	1028-42	Abnormal exocytotic release of glutamate in a mouse model of amyotrophic lateral sclerosis	116	6	2011	Glutamate	B6SJL	G93A Control	1	Synaptosomes were prepared from he whole spinal cord, cervical-thoracic (C1-T12) and thoracic-lumbar (T13-L6) regions of the cord or motor cortex

PMID	Author	Journal	Pages	Title	Vol	Iss	Year	Category	Gene Cross	Mouse Type	Figure	Methods
21521523	Fergani A, Eschbach J, Oudart H, Larmet Y, Schwalenstocker B, Ludolph AC, Loeffler JP, Dupuis L	Mol Neurodegener	26	A mutation in the dynein heavy chain gene compensates for energy deficit of mutant SOD1 mice and increases potentially neuroprotective IGF-1.	6	1	2011	IGF-1		G93A Control	5	Protein expression in spinal cord tissue
21726643	Caioli S, Curcio L, Pieri M, Antonini A, Marolda R, Severini C, Zona C	Neurobiol Dis	92-101	Substance P receptor activation induces downregulation of the AMPA receptor functionality in cortical neurons from a genetic model of Amyotrophic Lateral Sclerosis.	44	1	2011	GluR1	B6SJL	G93A Control	8	Protein expression in cultured cortical cells
22591194	Kalmar B, Edet-Amana E, Greensmith L	Amyo-troph Lateral Scler	378-92	Treatment with a coinducer of the heat shock response delays muscle denervation in the SOD1-G93A mouse model of amyotrophic lateral sclerosis.	13	4	2012	ChAT	C57BL/(	G93A Control	3 & 4	Radiochemical analysis of muscle, sciatic nerve and spinal cord tissues
23280929	Benkler C, Ben-Zur T, Barhum Y, Offen D	Glia	312-26	Altered astrocytic response to activation in SOD1(G93A) mice and its implications on amyotrophic lateral sclerosis pathogenesis.	61	3	2013	GLT-1	C57BL/(	G93A Control	2 & 3	Astrocyte primary cultures were prepared from the cortex of newborn mouse
23364798	Morel L, Regan M, Higashimori H, Ng SK, Esau C, Vidensky S, Rothstein J, Yang Y	J Biol Chem	7105-16	Neuronal exosomal miRNA-dependent translational regulation of astroglial glutamate transporter GLT1.	288	10	2013	GLT-1		G93A Control	3	Protein levels in astrocyte cultures
23673277	Song L, Gao Y, Zhang X, Le W	Neuroscience	281-90	Galactooligosaccharide improves the animal survival and alleviates motor neuron death in SOD1G93A mouse model of amyotrophic lateral sclerosis.	246		2013	TNF-alpha	C57BL/(	G93A Control	6	Western blot assay from spinal cord tissues
23752092	Caioli S, Pieri M, Antonini A, Guglielmotti A, Severini C, Zona C	Neuro-pharma-cology	247-60	Monocyte Chemoattractant Protein-1 upregulates GABA-induced current: evidence of modified GABAA subunit composition in cortical neurons from the G93A mouse model of Amyotrophic Lateral Sclerosis.	73		2013	GABA	B6SJL	G93A Control	1, 6, & 7	Primary dissociated cultures of cortical neurons and of spinal motor neurons from G93A mice
24041987	Albano R, Liu X, Lobner D	Exp Neurol	69-73	Regulation of system x(c)- in the SOD1-G93A mouse model of ALS.	250		2013	Glutamate	B6SJL	G93A Control	3	Experiments were performed using acute spinal cord slices from transgenic and littermate non-
25781653	Cai M, Choi SM, Yang EJ	Toxins (Basel)	846-58	The effects of bee venom acupuncture on the central nervous system and muscle in an animal hSOD1G93A mutant.	7	3	2015	TNF-alpha		G93A Control	3 & 5	Mice were sacrificed immediately after the behavioral testing, and the spinal cords were isolated for Western blotting.
25963727	Valbuena GN, Rizzardini M, Cimini S, Siskos AP, Bendotti C, Cantoni L, Keun HC	Mol Neurobiol	2222-40	Metabolomic Analysis Reveals Increased Aerobic Glycolysis and Amino Acid Deficit in a Cellular Model of Amyotrophic Lateral Sclerosis.	53	4	2016	Glutamate		G93A Control	2, 11, & 12	Mouse neuroblastoma N18TG2 × mouse embryonic spinal cord motor neurons expressing G93ASOD1 were co-cultured

PMID	Author	Journal	Pages	Title	Vol	Iss	Year	Category	Gene Cross	Mouse Type	Figure	Methods
26002422	Lee JK, Shin JH, Gwag BJ, Choi EJ	Neurobiol Dis	63-9	Iron accumulation promotes TACE-mediated TNF-alpha secretion and neurodegeneration in a mouse model of ALS.	80		2015	TNF-alpha	B6SJL	G93A Control	4	Levels blood serum of the mice were quantitated by ELISA
26648846	Jeyachandran A, Mertens B, McKissick EA, Mitchell CS. 2015;9:462. doi:10.3389/fncel.2015.00462.	Front Cell Neurosci		Type I Vs. Type II Cytokine Levels as a Function of SOD1 G93A Mouse Amyotrophic Lateral Sclerosis Disease Progression.	9	462	2015	TNF-alpha		G93A Control	3	Measures dealing with densities, mRNA or protein levels, and fold change for each of the cytokines
29553298	Tefera TW, Borges K	J Cereb Blood Flow Metab	1-15	Neuronal glucose metabolism is impaired while astrocytic TCA cycling is unaffected at symptomatic stages in the hSOD1(G93A) mouse model of amyotrophic lateral sclerosis.	21		2018	Glutamate,	B6SJL	G93A Control	3	Tissue samples of cortex and spinal cord were homogenized with a sonicator
